# Ethanol extract from *Artemisia argyi* leaves inhibits HSV-1 infection by destroying the viral envelope

**DOI:** 10.1186/s12985-023-01969-5

**Published:** 2023-01-16

**Authors:** Ping Liu, Lishan Zhong, Ji Xiao, Yuze Hu, Tao Liu, Zhe Ren, Yifei Wang, Kai Zheng

**Affiliations:** 1 Institute of Biomedicine, College of Life Science and Technology, Guangdong Province Key Laboratory of Bioengineering Medicine, Key Laboratory of Innovative Technology Research on Natural Products and Cosmetics Raw Materials, Jinan University, Guangzhou, 510632 China; 2 Guangdong Provincial Biotechnology Drug & Engineering Technology Research Center, National Engineering Research Center of Genetic Medicine, National Engineering Research Centre for Modernization of Chinese Medicine, Guangzhou, 510632 China; 3 School of Pharmaceutical Sciences, Medical School, Shenzhen University, Shenzhen, 518060 China

**Keywords:** *Artemisia argyi*, Ethanol extract, HSV-1, ACV-resistance, Viral envelope

## Abstract

Herpes simplex virus type 1 (HSV-1) is a widely disseminated virus that establishes latency in the brain and causes occasional but fatal herpes simplex encephalitis. Currently, acyclovir (ACV) is the main clinical drug used in the treatment of HSV-1 infection, and the failure of therapy in immunocompromised patients caused by ACV-resistant HSV-1 strains necessitates the requirement to develop novel anti-HSV-1 drugs. *Artemisia argyi*, a Traditional Chinese Medicine, has been historically used to treat inflammation, bacterial infection, and cancer. In this study, we demonstrated the antiviral effect and mechanism of ethanol extract of *A. argyi* leaves (hereafter referred to as ‘AEE’). We showed that AEE at 10 μg/ml exhibits potent antiviral effects on both normal and ACV-resistant HSV-1 strains. AEE also inhibited the infection of HSV-2, rotavirus, and influenza virus. Transmission electron microscopy revealed that AEE destroys the membrane integrity of HSV-1 viral particles, resulting in impaired viral attachment and penetration. Furthermore, mass spectrometry assay identified 12 major components of AEE, among which two new flavones, deoxysappanone B 7,3ʹ-dimethyl ether, and 3,7-dihydroxy-3′,4ʹ-dimethoxyflavone, exhibited the highest binding affinity to HSV-1 glycoprotein gB at the surface site critical for gB–gH–gL interaction and gB-mediated membrane fusion, suggesting their involvement in inactivating virions. Therefore, *A. argyi* is an important source of antiviral drugs, and the AEE may be a potential novel antiviral agent against HSV-1 infection.

**Supplementary Information:**

The online version contains supplementary material available at 10.1186/s12985-023-01969-5.

## Introduction

Herpes simplex virus type 1 (HSV-1) is a widely spread double-stranded DNA virus that infects and establishes latency in the central nervous system. Acute infection of HSV-1 or its reactivation from latency can cause herpes simplex encephalitis (HSE) and long-term neuro-inflammatory damage [1, 2]. In addition, HSV-1 neurotropic infection is widely accepted as one of the empirical factors of neurodegenerative diseases (e.g., Alzheimer's disease, Parkinson's disease) [2]. Notably, HSE has a high mortality rate in immunocompromised patients without appropriate antiviral therapy (up to 70%) [3, 4]. Acyclovir (ACV) is the most clinically used nucleoside analog against HSV-1 infection. ACV inhibits the enzymatic activity of HSV thymidine kinase (TK), interrupting viral DNA replication. As a result, ACV-resistant HSV strains isolated from clinic patients are mostly TK-altered and TK-negative/-low producer mutants [5]. Therefore, novel antiviral drugs are needed to treat HSV-1 infection-related diseases.

*Artemisia argyi* L. (Chinese mugwort) is a traditional Chinese medicinal herb with medicinal and food properties [6]. *A. argyi* has been historically used to warm menstruation, relieve pain, disperse dampness and cold, and regulate Qi and blood. Up to now, several volatile oils, terpenoids, flavonoids, alkaloids, and polysaccharides have been isolated from *A. argyi* extract, which demonstrates antioxidative [7], anti-cancer [8, 9], anti-inflammatory [10], antibacterial [11, 12] and immunosuppressive [13] effects. Notably, most studies focus on the aqueous extract and essential oil component of *A. argyi*. The biological activity of the non-oil components or the ethanolic extract is still unclear. One example was that *A. argyi* ethanolic extract alleviates inflammatory bowel disease and enhances immunomodulatory effects in lymphoid tissues [14]. Another recent work showed that ethanol extract of *A. argyi* possesses insecticidal activity against cabbage aphid (*Brevicoryne brassicae* L.) [15]. Therefore, investigating other potential functions of A. argyi ethanol extract is worthwhile*.*

Herein, we demonstrated the antiviral activity of a specific fraction from the ethanol extract of *A. argyi* (referred to as AEE). We found that AEE exhibits antiviral activity toward ACV-resistant and wild-type HSV-1 strains and HSV-2, rotavirus (RV), and influenza virus. Moreover, the antiviral mechanism was investigated using viral plaque assay, liquid chromatography/mass spectrometry assay, transmission electron microscope (TEM), and molecular docking. Our work suggests that AEE may be a potent agent against HSV-1 infection.

## Materials and methods

### Preparation of* A. argyi* leaves ethanol extract

*A. argyi* leaves were harvested in Tangyin country (Henan, China) and were air-dried under shade at room temperature. After drying, the plant samples were ground into powder, and two kilograms (2 kg) of leave powders were extracted three times by maceration with 95% ethanol for 24 h. The combined ethanol extract (252.3 g) was extracted successively with ethyl acetate (1 L), water, dichloromethane (1 L), and petroleum ether (1 L). The ethyl acetate-soluble fraction (12 g) and a dichloromethane-soluble fraction (35 g) were combined, separated by a silica gel column (800 g), and eluted with different ratios of methylene chloride: MeOH solvents (from 100:0 to 1:1) to yield 9 major fractions (fraction 1–9) (Additional file 1: Fig. S1). Fractions were finally freeze-dried and stored at − 20 °C for further usage. The anti-HSV-1 activities of fractions 1–9 were screened by the viral cytopathic effect (CPE) method. Briefly, Vero cells were treated with different concentrations of fraction 1–9 and HSV-1 (MOI = 0.1) for 48 h. The cytopathic changes were observed. No cytopathic lesion was recorded as "−", cytopathic lesions 1% ~ 25% as "+", cytopathic lesions 26%–50% as "++", cytopathic lesions 51% ~ 75% as "+++", and cytopathic lesions 76% ~ 100% as " +++++ ". Fraction-6 exhibited the highest anti-HSV-1 activity, which was further used for antiviral research (hereafter referred to as "**AEE or AEE (Fr.6)**, fraction-6 of ***A. argyi*** **E**thanol **E**xtract").

### Cells, viruses, and antibodies

Kidney epithelial cells MA-104 (CRL-2378.1) and Vero (CCL-81), neuronal cells SH-SY5Y (CRL-2266) and U87-MG (HTB-14) were obtained from the American Type Culture Collection Center (ATCC, USA). U87-MG, Vero, and SH-SY5Y cells were cultured in Dulbecco's modified Eagle's medium (8118305, GIBCO, USA), supplemented with 10% fetal bovine serum (FND500, ExCell Bio, Shanghai, China). MA-104 cells were cultured in modified Eagle's medium (A1451801, GIBCO, USA).

HSV-1 F strain (ATCC, USA) was grown in Vero cells and stored at − 80 °C for further use as previously described [16]. ACV-resistant strains HSV-1/153 and HSV-1/Blue were obtained from the Guangzhou Institutes of Biomedicine and Health (Guangzhou, China). HSV-1 F strain tagged by Green fluorescent protein (GFP) (GFP-HSV-1) was obtained from Professor Yuan Li of Jinan University (Guangzhou, China). HSV-2 and RV virus were obtained from the Wuhan Institute of Virology (Wuhan, China). As previously described, the influenza virus H1N1 was grown and stored at − 80 °C for further use [17]. Antibody anti-ICP0 (ab6513) and anti-gB (ab6506) were purchased from Abcam (Cambridge, UK), and anti-GAPDH (2118) was purchased from Cell Signaling Technology (Danvers, MA, USA).

### Cytotoxicity assay

Different cells were seeded in a 96-well plate (1 × 10^4^ cells/well) for 24 h and were then treated with different concentrations of AEE (0–80 μg/ml) for another 48 h. The cell viability was then evaluated by CCK8-kit (96992, Sigma-Alarich). The cells were added with 5 μl of CCK8 reagent and incubated at 37 °C for 1–2 h. A microplate reader was used to detect the OD value at 490 nm.

### Viral plaque assay

A viral plaque assay was used to examine the antiviral effect of AEE as previously described [16]. Briefly, Vero cells were seeded in a 24-well plate for 24 h before being infected with HSV-1 (MOI = 1). The virus was incubated at 37 °C for 2 h to promote viral particle absorption. The maintenance medium containing 1% methylcellulose (SIJIA BIOTECH, Guangzhou, China), with or without AEE, was used to replace the cell culture medium. After 72 h incubation, 10% formalin was used to fix the cells, and 1% crystal violet (Beyotime, Suzhou, China) was subsequently used to stain the cells. Finally, the total plaque numbers of each well were counted to calculate the inhibitory ratio accordingly.

### Virus titration assay

Virus titration was determined according to the HSV-1-induced cytopathic effects. Vero cells were treated with a diluted culture medium containing different concentrations of HSV-1 viral particles. The cells were then incubated at 37 °C for 48 h, and the 50% tissue culture infectious dose (TCID_50_) was measured according to the cellular cytopathic effects. The plaque-forming units (PFU)/ml were further converted from the TCID_50_ value.

### Quantitative real-time PCR (RT-qPCR)

Total RNA was extracted using the TRIZOL reagent (TIANGEN, Beijing, China) under the manufacturer's instructions. The PrimeScript RT Reagent Kit (TAKARA, Dalian, China) then transcribed the sample RNA (1 μg) into cDNA reversely. A Bio-Rad CFX96 Real-time PCR System (Bio-Rad) was used to determine the mRNA expression of different viral genes, and the internal reference gene *gapdh* was used to normalize gene mRNA level. All primer sequences are shown in Additional file 1: Table S1.

The DNA copy numbers of HSV-1 viral genes were collected and analyzed as before [16]. Briefly, cells were infected with HSV-1 with or without AEE for 24 h, and the cell pellets and supernatant were collected. The viral samples were frozen at − 80 °C and thawed three times. A UNIQ-10 viral DNA extraction kit (Sangon, China) was used to isolate total virus genomic DNA, which was further subjected to RT-qPCR to determine the DNA copy numbers of viral genes.

### Viral attachment, penetration, and inactivation assay

To evaluate the effect of AEE on viral attachment, Vero cells were pre-cooled at 4 °C for 1 h, then treated with AEE and HSV-1 (30 PFU/well) at 4 °C to facilitate viral particle attachment. After 80 min incubation, the culture medium was removed, and the cells were then incubated with a cover layer at 37 °C for another 72 h. Finally, the cells were fixed and stained to calculate the plaque numbers.

To evaluate the effect of AEE on viral penetration, Vero cells pre-cooled at 4 °C were incubated with HSV-1 (30 PFU/well) for 2 h at 4 °C. The culture medium was then replaced with an AEE-containing medium and cultured at 37 °C for another 10 min. The cells were washed with PBS buffer (pH = 3) and alkaline PBS (pH = 11.0) to remove viral particles not entering the cells. The cells were cultured with a cover layer at 37 °C for 72 h, and a plaque assay was performed.

To evaluate the effect of AEE on viral inactivation, HSV-1 viral particles (30–50 PFU/well) were treated with AEE for 2 h at 37 °C. Vero cells in a 24-well plate were then incubated with the virus-AEE mixture at 37 °C for another 2 h. A cover layer was used to replace the mixture and cultured at 37 °C for 72 h, which was then analyzed by plaque assay.

To evaluate the effect of AEE on the entry of RV, pre-cooled MA-104 cells were treated with RV (MOI = 1) and AEE (10 μg/ml) at 4 °C for 80 min. The culture medium was removed, and the cells were incubated at 37 °C for 24 h. Total RNA was extracted, and the mRNA expression levels of RV gene VP4 and VP7 were detected by RT-qPCR.

### Western blot assay

RIPA buffer (P0013B, Beyotime, China) containing 1 mM phenylmethylsulfonyl fluoride was used to extract total proteins. After measuring concentration, protein samples were separated by SDS-PAGE (10%-15% gradient) and were transferred to the appropriate polyvinylidene fluoride membrane (Millipore). The protein membrane was then blocked by 5% nonfat milk at room temperature for 1 h, incubated with different primary antibodies at 4 °C overnight, and then incubated with appropriate secondary antibodies for 1 h at room temperature. Viral proteins were further detected by ECL solutions, and protein bands were captured by a 5200-image analysis system (Tannon, Shanghai, China).

### LC–MS analysis

An ultra-high-performance liquid chromatograph (UltiMate 3000 UPLC system, Thermo, USA)/mass spectrometry (TripleTOF™ 5600 LC/MS, AB SCIEX, USA) was used to isolate the possible component of AEE. Software Analyst TF 1.7 and MS-DIAL 4.24 were used to acquire and analyze data. An Acquity UPLC HSS T3 analytical column (100 × 2.1 mm, 1.8 μm) was used for chromatographic separation, with gradient elution consisting of water with 0.1% formic acid (v/v, solvent A) and 0.1% formic acid in acetonitrile (solvent B) (positive ion mode), or consisting of water with 2 mM ammonium acetate (solvent A) and acetonitrile (solvent B) (negative ion mode).

### Molecular docking

Auto Dock 4.2.6 software (The Scripps Research Institute) was used to perform molecular docking assay. Briefly, the two-dimensional structures of 12 AEE components were obtained from the PubChem database (https://pubchem.ncbi.nlm.nih.gov/) and were further transformed into the PDB format by Chem3D software (Chem3D 18.0.0.231). The X-ray crystal structures of gD (PDB ID: 3U82), nectin-1 (PDB ID: 3SKU), gB (PDB ID: 4BOM), and PILRα (PDB ID: 5XO2) were downloaded from the PDB database (http://www.rcsb.org/). After the ligands and receptor molecule files were prepared, a grid box was set for the movement and rotation of the ligand via Glide (Maestero version 12.5, Schrodinger). AutoDock4 program was then run to process molecular docking. PyMOL 2.5 (DeLano Scientific LLC, CA, USA) was used to visualize the simulation. Finally, the PLIP (https://plip-tool.biotec.tu-dresden.de/) was used to analyze the ligand and receptor binding of hydrophobic bonds and hydrogen.

### Transmission electron microscopy

The morphology and shape of HSV-1 particles were examined by FEI Tecnai G2 Spirit TWIN transmission electron microscopy (TEM). Isolated viral particles in a volume of 1 ml medium were incubated with or without AEE (10 μg/ml) at 37 °C for 2 h, and the virus-AEE mixtures were prepared by dripping on a carbon-coated copper grid for subsequent procedures according to the instructions.

### Statistical analysis

Data were presented as mean ± SD. At least 3 independent experiments were performed. The GraphPad Prism 7 software was used for statistical analysis. One-way ANOVA or Student's *t*-test was carried out, with significance as ns, not significant, *P < 0.05, **P < 0.01, and ***P < 0.001.

## Results

### Cytotoxicity of AEE

**Fig. 1:**
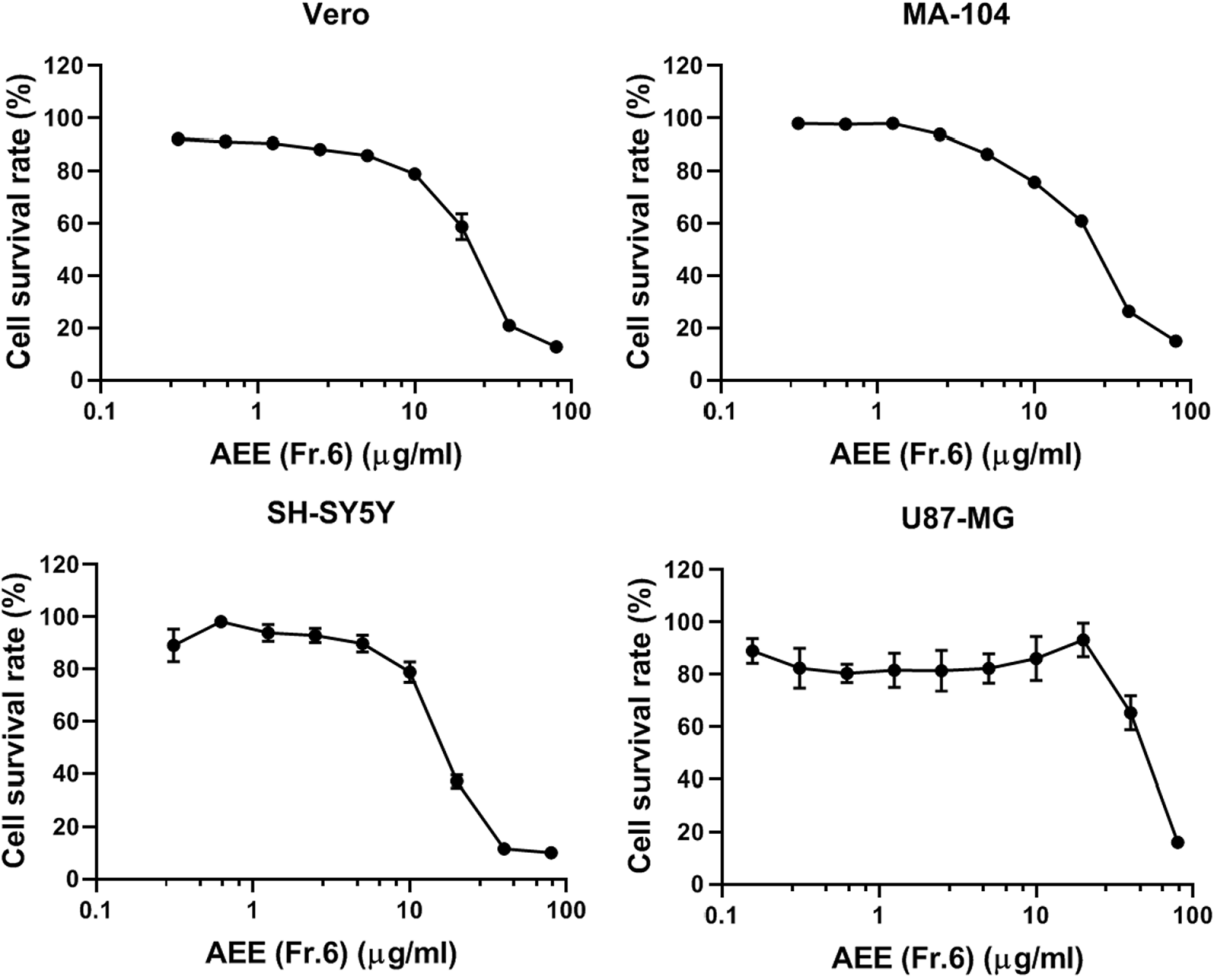
Cytotoxicity of AEE (Fr.6) on different cell lines. Cells were treated with different concentrations of AEE (Fr.6) for 48 h, and cell viability was determined by CCK-8 Kit. Data were presented as the means ± SD (n = 3)

The cytotoxicity of AEE on various HSV-1 host cells, including epithelial cells (Vero and MA-104) and neuronal cells (SH-SY5Y and U87-MG), were tested (Fig. 1). Cells were incubated with different concentrations of AEE (0–80 μg/ml) for 48 h, and the cell viability was analyzed via CCK-8 Kit. The CC_50_ concentration (CC_50_) of AEE toward epithelial cells (Vero and MA-104) and neuronal cells (SH-SY5Y and U87-MG) were 21.47 μg/ml, 23.35 μg/ml, 16.42 μg/ml, and 49.18 μg/ml, respectively. We found that no more than 15% of cell viability was reduced by 10 μg/ml AEE in all cell lines, and a concentration of AEE below 10 μg/ml was therefore used for the subsequent antiviral research.

### AEE inhibits HSV-1 infection

**Fig. 2:**
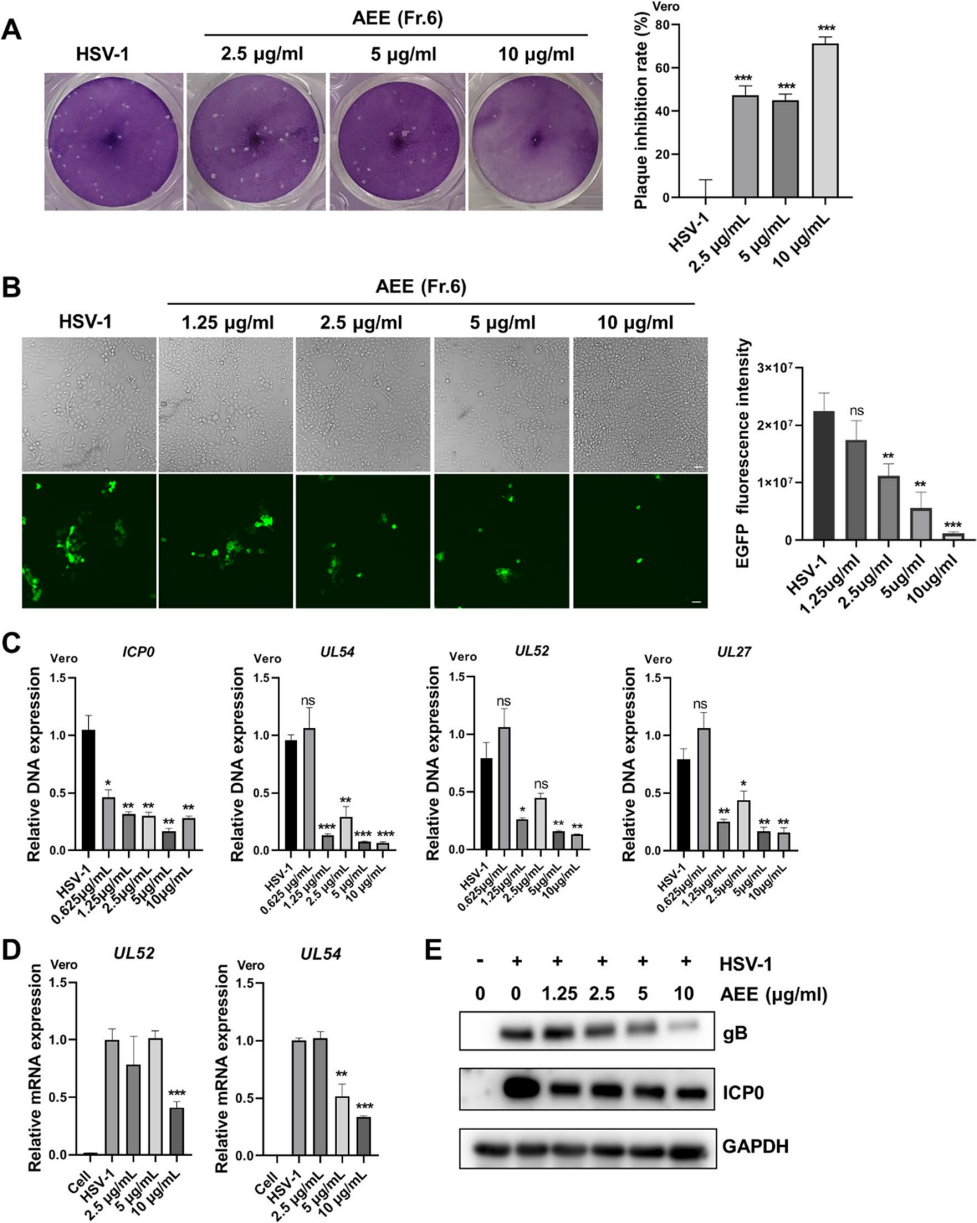
AEE exhibits potent anti-HSV-1 activity. **A** Vero cells were infected with HSV-1 (MOI = 0.1) for 2 h in the presence of AEE (10, 5, 2.5 μg/ml) and were then subjected to plaque assays (left). The inhibitory rate was calculated according to the number of plaques (right). **B** Vero cells were infected with EGFP-HSV-1 (MOI = 0.1) for 24 h in the presence of AEE (10, 5, 2.5, 1.25 μg/ml), and fluorescent images were captured by microscopy (left). GFP fluorescence intensity was calculated by Image J software (right). Scale bar, 100 μm. **C** and **D** Vero cells were infected with HSV-1 (MOI = 0.1) in the presence of AEE for 24 h, and total DNA or RNA was extracted. The DNA copy numbers (**C**) or the mRNA expression levels (**D**) of viral genes were analyzed by RT-qPCR. **E** Vero cells were infected with HSV-1 (MOI = 1) for 24 h in the presence of AEE, and total protein was extracted for western blot analysis of viral protein ICP0 and gB. Data were presented as the means ± SD (n = 3). *P < 0.05, **P < 0.01, ***P < 0.001 versus HSV-1-treated group, unpaired t test

The anti-HSV-1 activity of AEE was first examined on epithelial Vero cells via viral Plaque assay (Fig. 2A), which showed that the viral plaque number was largely decreased by 2.5 μg/ml of AEE. Besides, the infectious efficacy of GFP-tagged HSV-1 was tested upon AEE treatment. As shown in Fig. 2B, AEE significantly reduced the GFP-HSV-1-infected cell numbers, restored cell proliferation, and decreased the immunofluorescent intensity of GFP. In addition, RT-qPCR analysis showed that AEE markedly reduced the DNA copy numbers of viral late gene *UL27,* early gene *UL52,* and immediate early gene (*ICP0* and *UL54*) (Fig. 2C), as well as the mRNA expression of *UL52* and *UL54* (Fig. 2D)*.* Furthermore, AEE significant decreased the protein levels of HSV-1 glycoprotein gB and early protein ICP0 in a concentration-dependent manner (Fig. 2E).

**Fig. 3:**
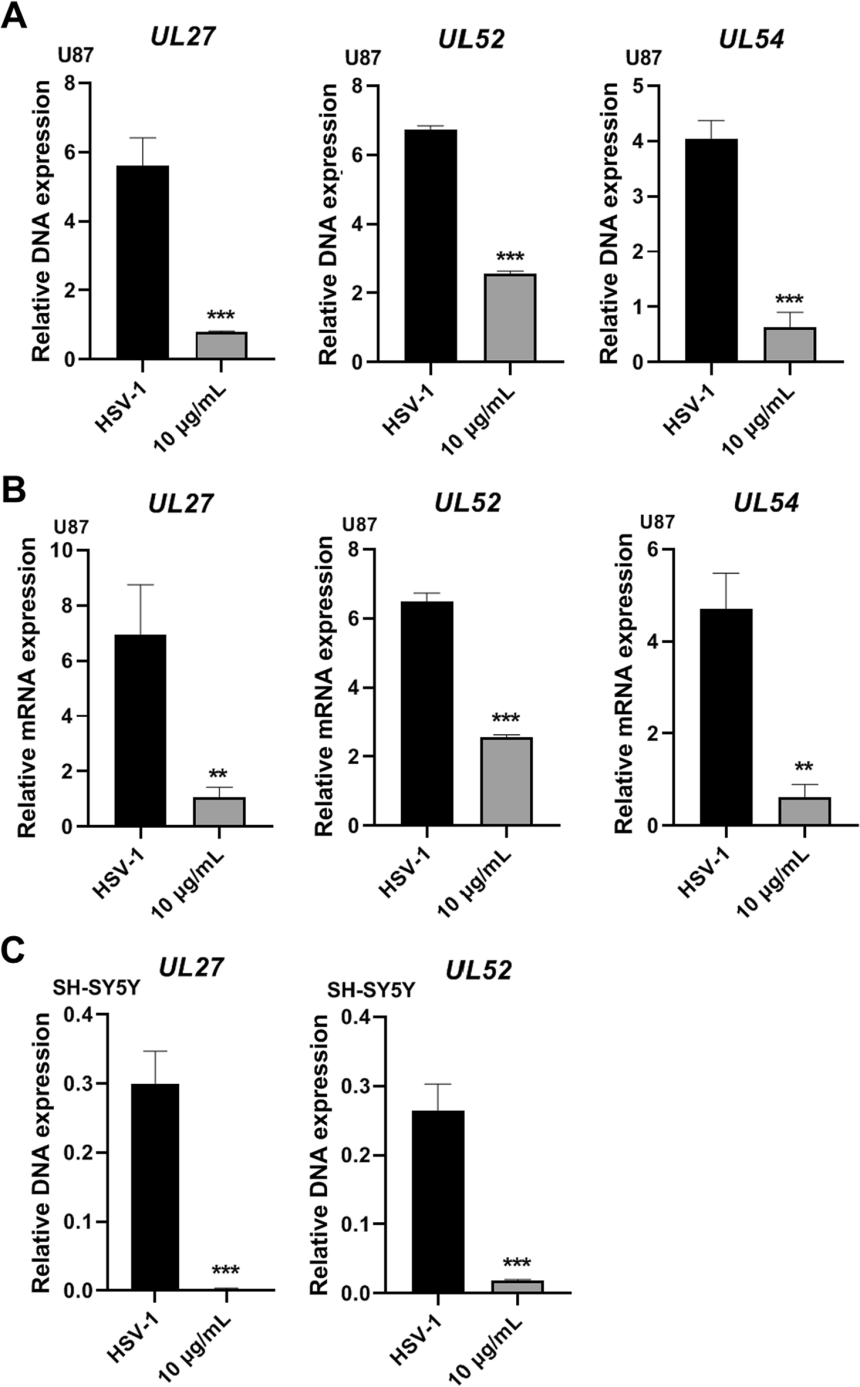
Anti-HSV-1 effect of AEE on neuronal cells. **A** and **B** U87-MG cells were infected with HSV-1 (MOI = 0.1) in the presence of AEE (10 μg/ml) for 24 h, and total DNA or RNA was extracted. The DNA copy numbers (**A**) or the mRNA expression levels (**B**) of viral genes were analyzed by RT-qPCR. **C** SH-SY5Y cells were infected with HSV-1 (MOI = 0.1) in the presence of AEE (10 μg/ml) for 24 h, and the DNA copy numbers of viral genes were determined by RT-qPCR. Data were presented as the means ± SD (n = 3). *P < 0.05, **P < 0.01, ***P < 0.001 versus HSV-1-treated group, unpaired t test

We also examined the antiviral effects of AEE on neuronal cells (U87-MG and SH-SY5Y cells). Similarly, AEE treatment inhibited the DNA copy numbers and mRNA expression of different viral genes (*UL54*, *UL52,* and *UL27*) in U87-MG cells (Fig. 3A and B). AEE also reduced the DNA copy numbers of viral genes in SH-SY5Y cells (Fig. 3C). These results indicate the strong antiviral effect of AEE against wild-type HSV-1 infection.

### AEE inhibits the infection of ACV-resistant HSV-1 strains

**Fig. 4:**
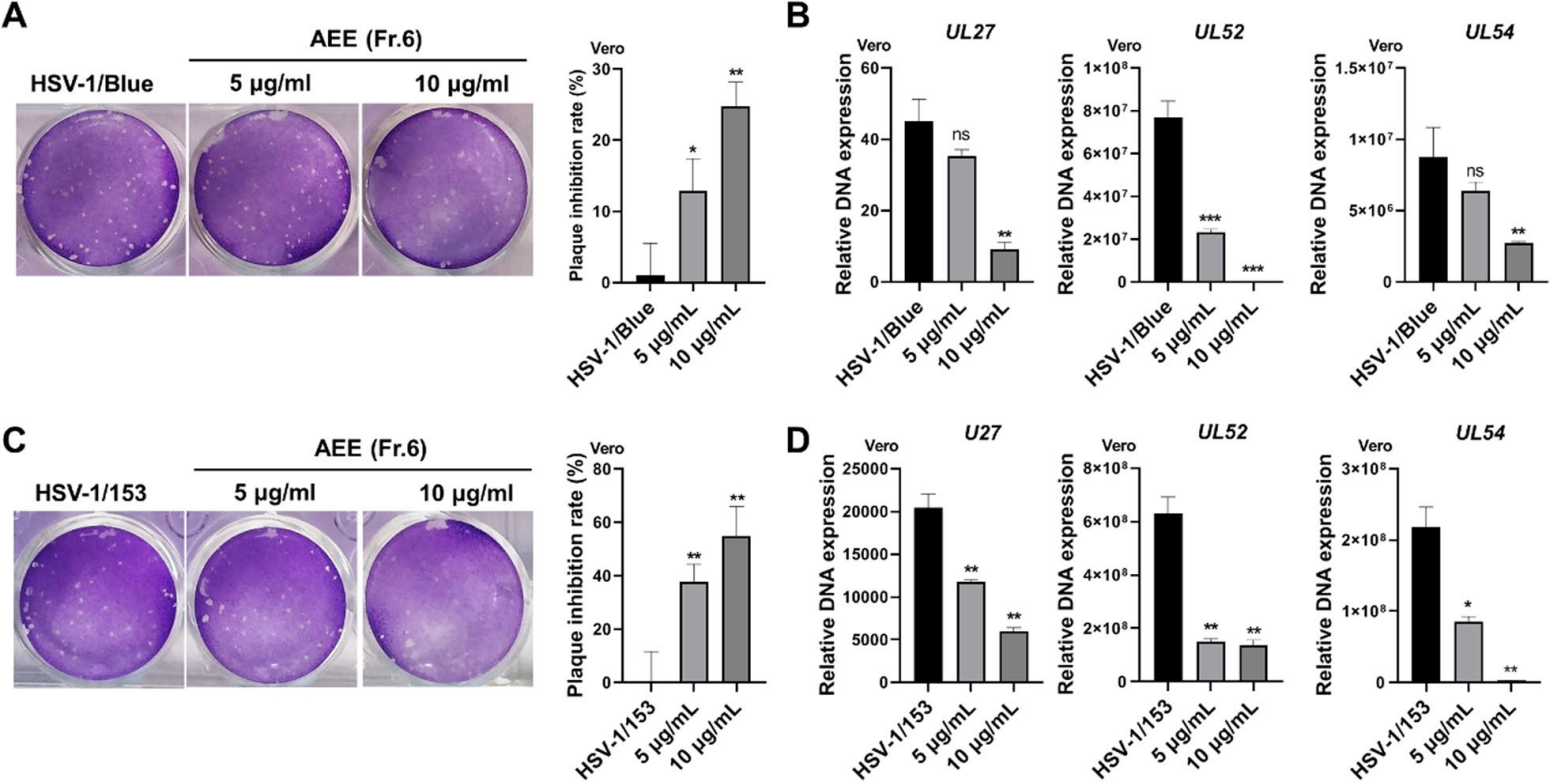
AEE inhibits ACV-resistant strains. **A** and **C** Vero cells were infected with HSV-1/Blue (**A**) or HSV-1/153 (**C**) (MOI = 0.1) for 2 h in the presence of AEE and were then subjected to plaque assays (left). The inhibitory rate was calculated according to the number of plaques (right). **B** and **D** Vero cells were infected with HSV-1/Blue (**B**) or HSV-1/153 (**D**) (MOI = 0.1) in the presence of AEE for 24 h, and the DNA copy numbers of viral genes were determined by RT-qPCR. Data were presented as the means ± SD (n = 3). *P < 0.05, **P < 0.01, ***P < 0.001 versus HSV-1-treated group, unpaired t test

Next, we assessed whether AEE could inhibit the infection of ACV-resistant strains (HSV-1/153 and HSV-1/Blue) [16]. Vero cells were challenged with HSV-1/153 or HSV-1/Blue in the presence or absence of AEE for 2 h, and plaque assays were performed. As shown in Fig. 4A and C, AEE at 10 μg/ml exhibited 30% and 60% inhibitory rates against HSV-1/153 and HSV-1/Blue, respectively. In addition, RT-qPCR analysis demonstrated that AEE significantly reduced the DNA copy numbers of viral genes of both ACV-resistant strains (Fig. 4B and D). Therefore, AEE can inhibit the infection of ACV-resistant HSV-1 strains.

### AEE inhibits the infection of HSV-2, RV, and influenza virus

**Fig. 5:**
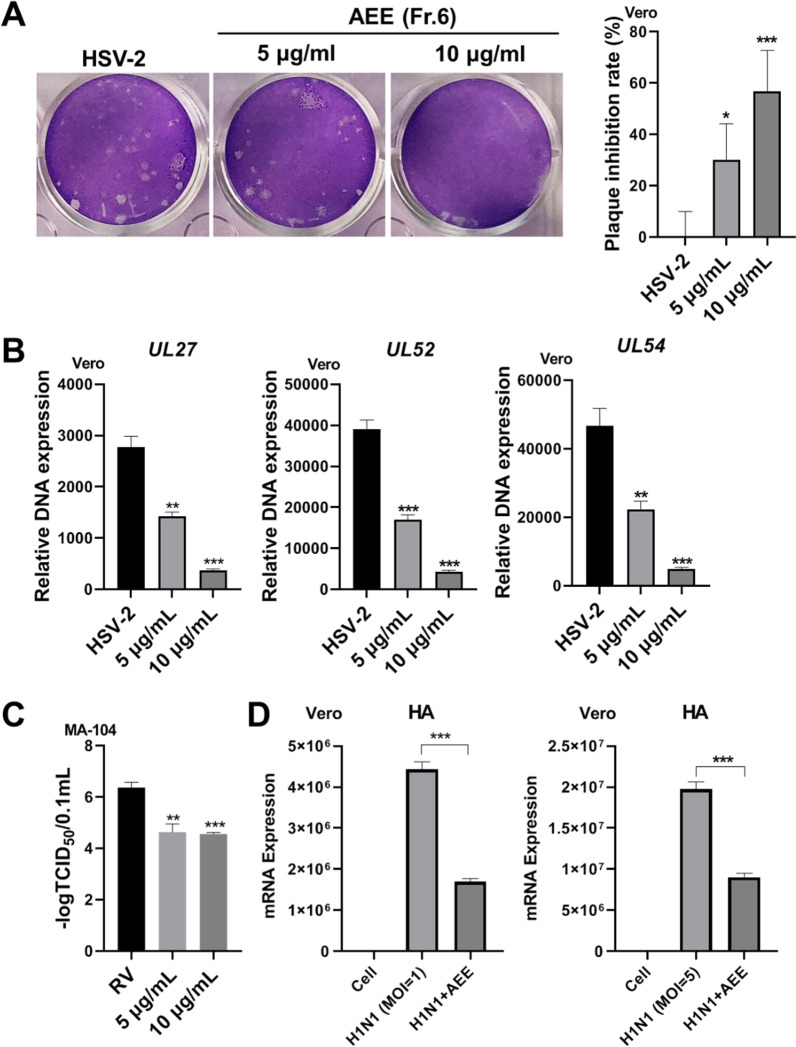
AEE inhibits HSV-2 and RV infection. **A** Vero cells were infected with HSV-2 (MOI = 0.1) for 2 h in the presence of AEE, and a viral plaque assay was performed (left). The inhibitory rate was calculated according to plaque numbers (right). **B** Vero cells were infected with HSV-2 (MOI = 0.1) in the presence of AEE for 24 h, and the total viral DNA was extracted to perform RT-qPCR. **C** MA-104 cells were infected with rotavirus (RV) (MOI = 0.1) for 72 h in the presence of AEE, and the virus titer was determined. Data were presented as the means ± SD (n = 3). **D** Vero cells were infected with H1N1 (MOI = 1 or 5) in the presence of AEE (10 μg/ml) at 37 °C for 24 h. Total RNA was extracted, and the mRNA expression of the H1N1 gene HA was detected by RT-qPCR. *P < 0.05, **P < 0.01, ***P < 0.001 versus virus group, unpaired t test

To investigate whether AEE exerts a virus-specific antiviral activity, we examined the effect of AEE on other viruses, including HSV-2 (the other serotype of alphaherpesvirus), rotavirus (RV) (a non-enveloped double-stranded RNA virus that causes severe diarrhea among infants and young children), and influenza virus. Notably, plaque assay clearly showed that AEE reduced the viral plaque number of HSV-2 (Fig. 5A). RT-qPCR assay also demonstrated that AEE significantly inhibited the DNA copy numbers of HSV-2 genes (Fig. 5B). In addition, viral titers of RV on MA-104 cells were measured, which was still largely decreased by AEE treatment (Fig. 5C). Furthermore, AEE treatment largely reduced the mRNA expression of influenza virus H1N1 gene HA (Fig. 5D). Together, these results indicate that AEE inhibits the infection of HSV-2, RV and influenza virus.

### AEE destroys envelope integrity to inhibit HSV-1 early infection

**Fig. 6:**
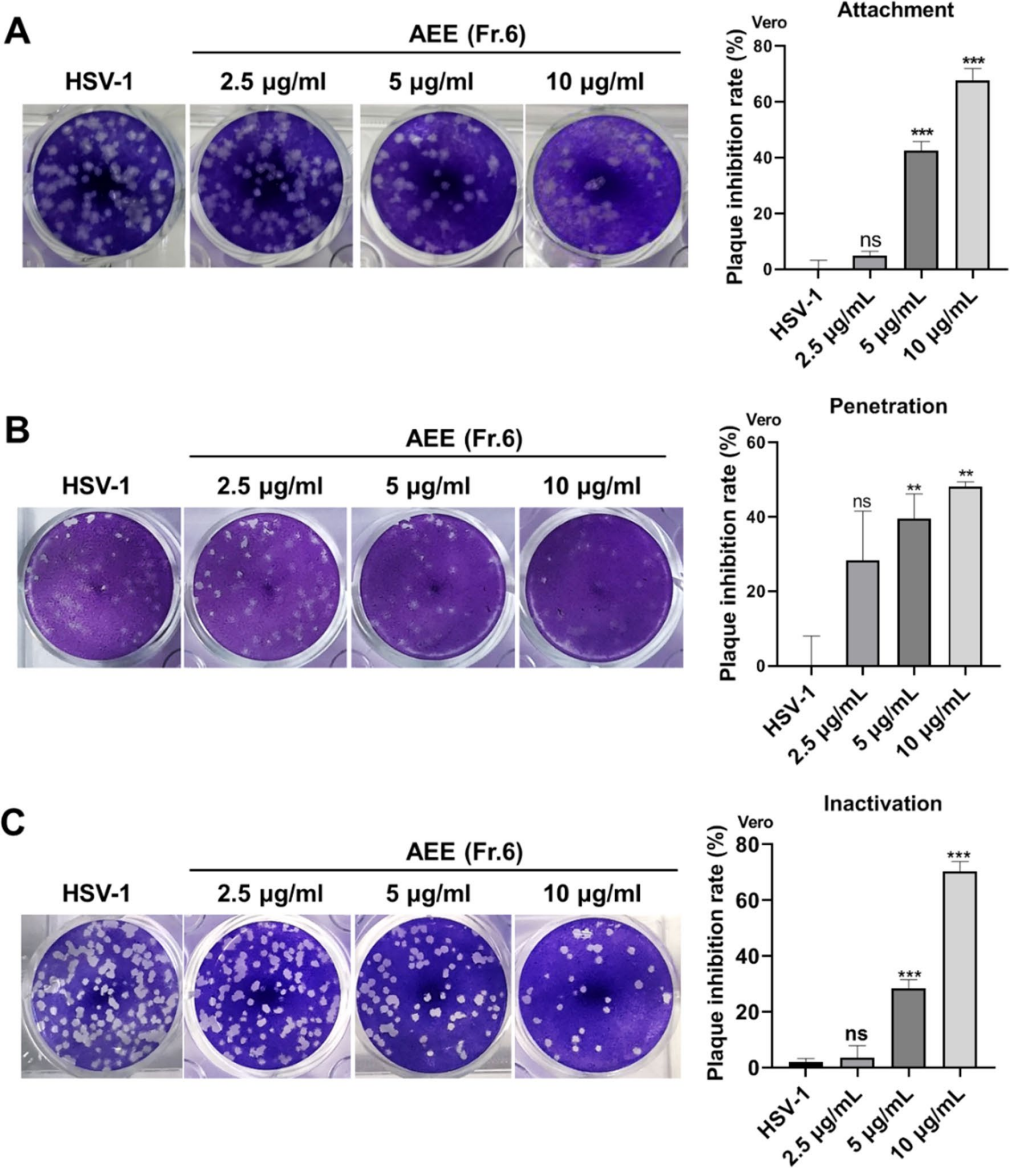
AEE inhibits HSV-1 early infection process. **A** AEE inhibited HSV-1 attachment. Vero cells were pre-cooled at 4 °C for 1 h and were infected with HSV-1 (MOI = 0.1) in the presence of AEE at 4 °C for 80 min. The cells were then incubated at 37 °C for 72 h and subjected to plaque assays. **B** AEE inhibited viral penetration. Pre-cooled Vero cells were treated with HSV-1 (MOI = 0.1) and AEE at 4 °C for 2 h, then incubated at 37 °C for another 10 min. After incubation, the bound but not entered virions were removed by alkaline PBS buffer (pH = 11.0). The cells were then subjected to plaque assay. **C** AEE directly inactivated viral particles. HSV-1 Virions were incubated with AEE at 37 °C for 2 h. After incubation, the drug-virus mixture was added to Vero cells to perform a plaque assay. The inhibitory rate was presented as the means ± SD (n = 3). *P < 0.05, **P < 0.01, ***P < 0.001 versus virus group, unpaired t test

To further determine the possible antiviral mechanism of AEE on HSV-1 infection, we tested the inhibitory effect of AEE on HSV-1 early infection processes, such as viral penetration and attachment. By plaque assay, we found that AEE significantly affected viral attachment (Fig. 6A) and penetration (Fig. 6B). More importantly, incubation of AEE with HSV-1 virions for 2 h markedly impaired the infectious ability of HSV-1, implying that AEE directly inactivated viral particles to prevent viral early infection (Fig. 6C).

**Fig. 7:**
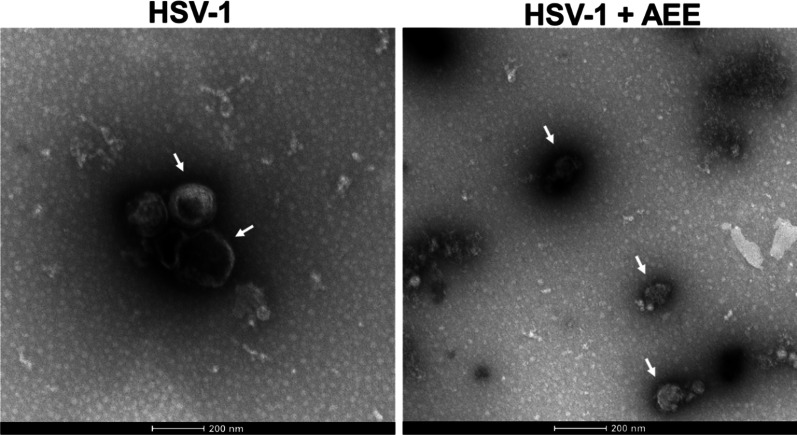
AEE destroys the HSV-1 envelope. White arrows indicated the intact spherical structure and envelope of HSV-1 viral particles (left) or the AEE-induced destruction of the HSV-1 envelope (right)

Furthermore, we used transmission electron microscopy (TEM) to observe the entitle structure of HSV-1virion, which revealed an intact and smooth viral envelope in the HSV-1 group (Fig. 7). On the contrary, apparently smaller and irregular viral particles were seen in AEE-treated group (indicated by arrows), which had an incomplete viral envelope with unclear boundaries and obvious gaps. Finally, we investigated whether AEE prevents the entry of non-enveloped virus (e.g., RV), and found that AEE does not affect the entry of RV, as evidenced by the unchanged viral gene expression (Additional file 1: Fig. S2). It is reasonable that AEE influences other infection stages of RV, such as viral replication and release, to inhibit RV infection (Fig. 5C). Together, these results suggest that AEE directly destroys the intact envelope integrity to inactivate HSV-1 virion and inhibits HSV-1 attachment and penetration.

### AEE major components interact with HSV-1 glycoproteins

**Fig. 8:**
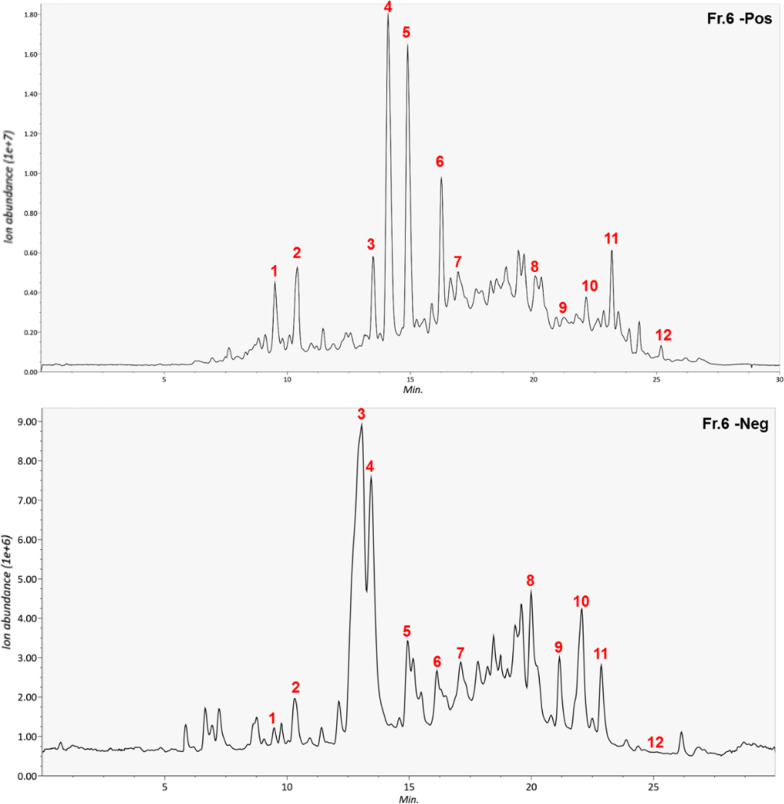
Major components of AEE determined by LC–MS. UV chromatograms of AEE in both positive and negative models were shown. The peak numbers of compounds are listed in Table 1

Finally, the LC–MS method was used to determine the possible components of AEE, and 12 major compounds were identified. The typical chromatogram is shown in Fig. 8, and specific information about the 12 major components is shown in Table 1. Several components, including two new flavones, deoxysappanone B 7,3′-dimethyl ether, and 3,7-dihydroxy-3′,4′-dimethoxyflavone, have never been documented, isolated, or purified to determine their biological activities.

**Table 1:** Identification of phytochemicals in AEE (fr. 6) using LC–MS

Peak	T_R_(min)	Precursor m/z	Reference m/z	Adduct	Area	Formula	Proposed Compound
1	9.628284	263.1273	263.1278	[M + H]+	1,477,670.1	C_15_H_18_O_4_	Parthenin
2	10.4298	315.1207	315.1227	[M + H]+	3,508,640.5	C_18_H_18_O_5_	Deoxysappanone B 7,3"-Dimethyl Ether
3	13.45003	303.086	303.0863	[M + H]+	3,904,619	C_16_H_14_O_6_	Hesperetin
4	14.11855	331.0804	331.0812	[M + H]+	121,957,608	C_17_H_14_O_7_	Quercetin 3,7-Dimethyl Ether M + H
5	15.87025	315.0862	315.0863	[M + H]+	10,475,061	C_17_H_14_O_6_	3,7-Dihydroxy-3′,4′-Dimethoxyflavone
6	16.87828	285.0763	285.0758	[M + H]+	2,161,221.3	C_16_H_12_O_5_	Acacetin
7	17.65963	221.1901	221.19	[M + H]+	2,346,044.3	C_15_H_24_O	Farnesal
8	20.30435	229.1228	229.122	[M + H]+	1,771,541.4	C_15_H_16_O_2_	12-Hydroxy-4,4-Bisnor-4,8,11,13-Podocarpatetraen-3-One
9	22.0197	255.2364	255.233	[M − H]−	15,856,229	C_16_H_32_O_2_	Isopalmitic Acid
10	22.49508	415.3193	415.3204	[M + H]+	1,159,666.8	C_27_H_42_O_3_	Diosgenin
11	23.5801	399.3264	399.3261	[M + H − 2O]+	1,028,603.9	C_27_H_44_O_3_	Calcitriol
12	25.18197	338.3438	338.3424	[M + H]+	5,165,575.5	C_22_H_43_NO	Erucamide

**Fig. 9:**
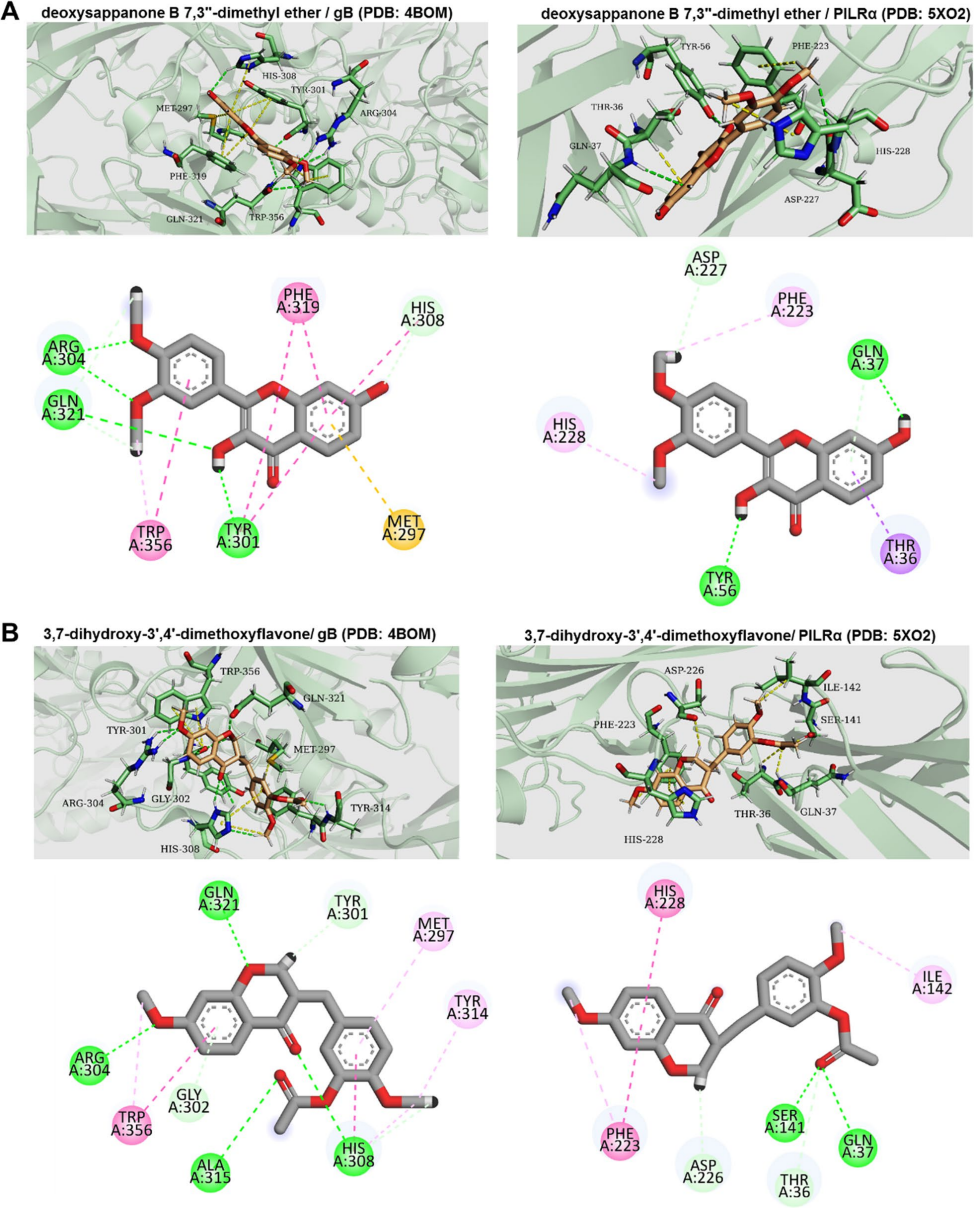
AEE components interact with HSV-1 gB and PILRα. **A** and **B** 3D schematic diagram of the binding of deoxysappanone B 7,3′-dimethyl ether (**A**) or 3,7-dihydroxy-3′,4′-dimethoxyflavone (**B**) to the binding site of gB (PDB: 4BOM) and PILRα (PDB: 5XO2), respectively. The interactive amino acids were also shown

According to the TEM result that AEE destroys the envelope integrity to inactivate HSV-1, we investigated whether AEE components directly interact with HSV-1 glycoprotein gB/gD or their corresponding receptors gB-PILRα/gD-nectin-1 to disrupt the spatial conformation change and the fusion with the host cell membrane. A molecular docking assay of 12 AEE components was performed, and the docking parameters were shown in Additional file 1: Table S2. There were no appropriate binding statuses of gD/gD-nectin-1 with these 12 AEE components. However, the two new flavones, deoxysappanone B 7,3′-dimethyl ether, and 3,7-dihydroxy-3′,4′-dimethoxyflavone, exhibited the lowest binding energies and the highest docking scores with gB and PILRα. The steady binding models of deoxysappanone B 7,3′-dimethyl ether and 3,7-dihydroxy-3′,4′-dimethoxyflavone with gB and gB receptor PILRα were shown in Fig. 9A and B, respectively. Notably, the two new flavones are tightly located at the site of the gB surface, where Gh–gL fusion peptides were bounded to promote gB fusion [18], implying that both flavones block the interaction between gB and gH–gL to prevent gB conformational change and fusion. Therefore, these results suggest that the two new flavones interact with HSV-1 gB to impair gB-mediated attachment and penetration.

## Discussion

Natural plants and functional foods are important sources for exploring novel antiviral drugs or candidates. With a long usage history, *A. argyi* functions as a dietary nutritional plant to provide various health benefits [6]. In this study, we added the antiviral activity to the broad spectrum function of *A. argyi*, and revealed that AEE, the specific fraction of *A. argyi* leaves ethanolic extract*,* inhibits the wild-type infection and ACV-resistant HSV-1 strains, as well as HSV-2, RV and influenza virus. We also found that AEE mainly inhibits viral attachment and penetration by directly destroying the envelope integrity of HSV-1 viral particles. Furthermore, 12 components of AEE were identified by LC–MS assay, and molecular docking assay indicated that two new flavones, deoxysappanone B 7,3′-dimethyl ether, and 3,7-dihydroxy-3′,4′-dimethoxyflavone, exhibit the highest binding affinity to HSV-1 glycoprotein gB at the surface site critical for gB–gH–gL interaction and gB-mediated membrane fusion, suggesting their involvement in inactivating virions. Therefore, AEE from *A. argyi* leaves represents a potential novel antiviral agent.

Although the bioactive ingredients of *A. argyi* exhibit various pharmacological and therapeutic functions, few studies have investigated the anti-infectious diseases of *A. argyi* [11, 12]. We presented here that AEE inhibits HSV-1 and HSV-2 infection and exerts inhibitory function against ACV-resistant HSV-1 strains, implying a novel antiviral mechanism different from ACV. Indeed, plaque assay and TEM analysis confirmed the different actions of AEE, which destroys the viral envelope to impair HSV-1 attachment and penetration (Figs. 7 and 8). On the contrary, AEE does not affect the entry of non-enveloped virus RV (Additional file 1: Fig. S2). It is interesting to examine whether AEE inhibits the infection of other enveloped viruses, such as Respiratory Syncytial Virus and Vesicular Stomatitis Virus, through disintegrating envelope and hindering envelope-mediated membrane fusion. Apart from this, AEE might affect other infection stages, such as viral replication and release, to inhibit RV infection (Fig. 5C). Considering the global pandemic caused by a respiratory virus, further works are inspired to investigate whether AEE still has potent antiviral activity against respiratory viruses, such as SARS-COV-2 and influenza virus [19].

In addition, 12 compounds, including two new flavones, deoxysappanone B 7,3′-dimethyl ether, and 3,7-dihydroxy-3′,4′-dimethoxyflavone, were identified as major components of AEE via LC–MS. Unfortunately, we have attempted to isolate each component of AEE but have failed due to the limitation of purification conditions and the much lower levels of each component in *A. argyi* leaves. Previous works have demonstrated that flavones from *A. argyi* leaves have anticoagulant [20], antimutagenesis [21], antioxidant [22, 23], and antitumor effects [24]. By molecular docking, we found that the two flavones bind more stably to HSV-1 gB at the surface site critical for gB–gH–gL interaction and gB-mediated membrane fusion [18]. It is, therefore, reasonable that these two flavones interrupt gB-mediated membrane fusion and damage viral envelope integrity, resulting in inhibited viral attachment and penetration. However, the two flavones had never been isolated before, and their biological functions remain to be clarified. Interestingly, analogs of the two flavones, deoxysappanone B 7,4′-dimethyl ether, deoxysappanone B 7,3′-dimethyl ether acetate, and 5,7-dihydroxy-3′,4′-dimethoxyflavone have been demonstrated to have anti-cryptosporidial [25], anti-angiogenic [26] or antibacterial [27] activity. Therefore, isolation and purification of the two new flavones from AEE to determine whether they have similar antibacterial or antiviral activities are needed in the future.

Altogether, our study uncovers an unappreciated role of ethanol extract of *A. argyi* in inhibiting herpesvirus infection via damaging viral envelope integrity to impair early viral infection. The *A. argyi* ethanolic extract and the newly identified flavones may provide novel approaches for mitigating HSV-1 infection.

## Supplementary Information

**Additional file 1.** **Figure S1**. Schematic illustration of the preparation of A. argyi Leaves Ethanol Extract. 9 major fractions (fraction 1-9) were obtained and their anti-HSV-1 activities were screened by CPE method. Vero cells were infected with HSV-1 (MOI=0.1) in the presence of different concentrations of fraction 1-9 for 48 h. The cytopathic changes were observed and recorded as follows: no cytopathic lesions, "-"; 1%~25% cytopathic lesions, "+"; 26%-50% cytopathic lesions, "++"; 51%~75% cytopathic lesions, "+++"; 76%~100% cytopathic lesions, "+++++". Fractions were finally freeze-dried and stored at -20°C until further use. Fraction-6 exhibited the highest anti-HSV-1 activity, which was further used for antiviral research (hereafter referred to as ′AEE or AEE (Fr.6), fraction-6 of A. argyi Ethanol Extract′). **Figure S2**. Effect of AEE on the entry of non-enveloped virus RV. Pre-cooled MA-104 cells were treated with RV (MOI = 1) and AEE (10 μg/ml) at 4°C for 80 min. The culture medium was then removed and the cells were incubated at 37°C for 24 h. Total RNA was extracted and the mRNA expression levels of RV gene VP4 and VP7 were detected by RT-qPCR. n.s, not significant versus virus group, unpaired t test. **Table S1**. Primer sequence used in this study. **Table S1**. Molecular docking results of 12 major components of AEE interacting with HSV-1 gB/gD and their receptors (Binding energy, kcal/mol).

## Data Availability

All data generated or analyzed during this study are included in this published article and its supplementary information files.
