# Supplementary material for: Ethanol extract from Artemisia argyi leaves inhibits HSV-1 infection by destroying the viral envelope
Source: Virol J. 2023 Jan 16;20:8. doi: 10.1186/s12985-023-01969-5 (PMC9841929; doi:10.1186/s12985-023-01969-5)
Supplement: Supplementary file 1 — Additional file 1. Figure S1. Schematic illustration of the preparation of A. argyi Leaves Ethanol Extract. 9 major fractions (fraction 1-9) were obtained and their anti-HSV-1 activities were screened by CPE method. Vero cells were infected with HSV-1 (MOI=0.1) in the presence of different concentrations of fraction 1-9 for 48 h. The cytopathic changes were observed and recorded as follows: no cytopathic lesions, "-"; 1%~25% cytopathic lesions, "+"; 26%-50% cytopathic lesions, "++"; 51%~75% cytopathic lesions, "+++"; 76%~100% cytopathic lesions, "+++++". Fractions were finally freeze-dried and stored at -20°C until further use. Fraction-6 exhibited the highest anti-HSV-1 activity, which was further used for antiviral research (hereafter referred to as ′AEE or AEE (Fr.6), fraction-6 of A. argyi Ethanol Extract′). Figure S2. Effect of AEE on the entry of non-enveloped virus RV. Pre-cooled MA-104 cells were treated with RV (MOI = 1) and AEE (10 μg/ml) at 4°C for 80 min. The culture medium was then removed and the cells were incubated at 37°C for 24 h. Total RNA was extracted and the mRNA expression levels of RV gene VP4 and VP7 were detected by RT-qPCR. n.s, not significant versus virus group, unpaired t test. Table S1. Primer sequence used in this study. Table S1. Molecular docking results of 12 major components of AEE interacting with HSV-1 gB/gD and their receptors (Binding energy, kcal/mol). [file 12985_2023_1969_MOESM1_ESM.docx]

# Supplementary information

**Ethanol Extract from *Artemisia argyi* Leaves inhibits HSV-1 infection by destroying the viral envelope**

# Supplementary Figures

**
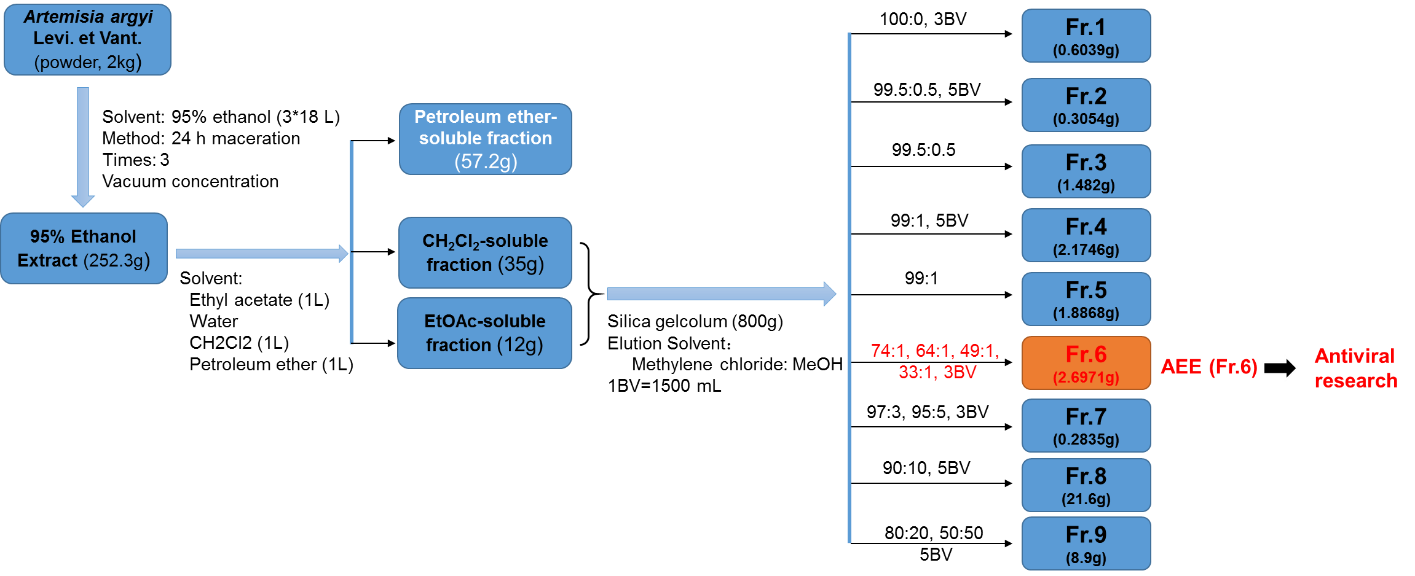
**

**Figure S1**. **Schematic illustration of the preparation of** *A. argyi* Leaves Ethanol Extract. 9 major fractions (fraction 1-9) were obtained and their anti-HSV-1 activities were screened by CPE method. Vero cells were infected with HSV-1 (MOI=0.1) in the presence of different concentrations of fraction 1-9 for 48 h. The cytopathic changes were observed and recorded as follows: no cytopathic lesions, "-"; 1%~25% cytopathic lesions, "+"; 26%-50% cytopathic lesions, "++"; 51%~75% cytopathic lesions, "+++"; 76%~100% cytopathic lesions, "+++++". Fractions were finally freeze-dried and stored at -20°C until further use. Fraction-6 exhibited the highest anti-HSV-1 activity, which was further used for antiviral research (hereafter referred to as ′**AEE or AEE (Fr.6)**, fraction-6 of ***A****. argyi* **E**thanol **E**xtract′).


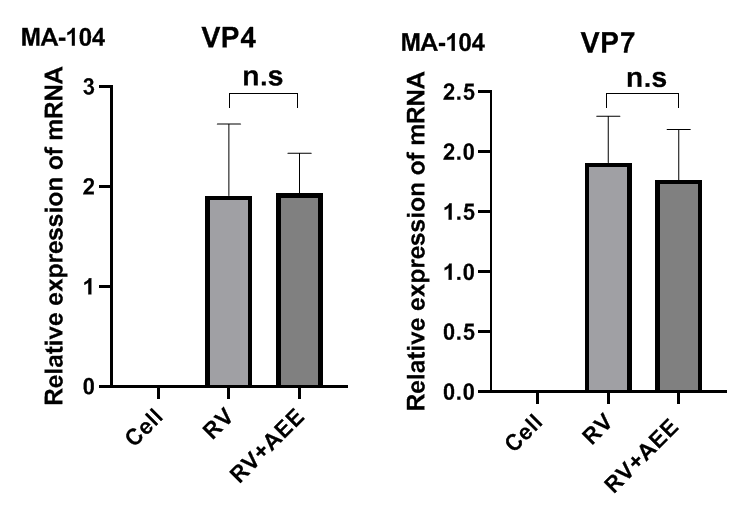


**Figure S2. Effect of AEE on the entry of non-enveloped virus RV.** Pre-cooled MA-104 cells were treated with RV (MOI=1) and AEE (10 μg/ml) at 4°C for 80 min. The culture medium was then removed and the cells were incubated at 37°C for 24 h. Total RNA was extracted and the mRNA expression levels of RV gene VP4 and VP7 were detected by RT-qPCR. n.s, not significant versus virus group, unpaired t test.

# Supplementary table

**Table S1**. Primer sequence used in this study

| Gene | Sequence (forward) | Sequence (Reverse) |
| --- | --- | --- |
| *gapdh* | CACCATCTTCCAGGAGCGAG | AGAGGGGGCAGAGATGATGA |
| *ICP0* | CCCACTATCAGGTACACCAGC | CTGCGCTGCGACACCTTTT |
| *ul54* | TGGCGGACATTAAGGACATTG | TGGCCGTCAACTCGCAGA |
| *ul52* | AGGCCATCAAGGACATCTGC | AATACGGCGCTCCACGTAAA |
| *Ul27* | GCCTTCTTCGCCTTTCGC | CGCTCGTGCCCTTCTTCTT |
| *VP4* | TGGCGTCCTCACTTTATCGG | CCCATGACGTCGGTGGTAAT |
| *VP7* | CTACAACGTGCACAATCCGA | ATTTGCGGAGAGGTTGTGGG |
| *HA* | TATTTGGAGCCATTGCCGGT | GATCCGCTGCATAGCCTGAT |

**Table S2.** Molecular docking results of 12 major components of AEE interacting with HSV-1 gB/gD and their receptors (Binding energy, kcal/mol)

| Ligand | gB | | | gB-PILRα | | | gD | | | gD-nectin-1 | | |
| --- | --- | --- | --- | --- | --- | --- | --- | --- | --- | --- | --- | --- |
|  | Docking score | Glide gscore | MMGBSA dG Bind | Docking score | Glide gscore | MMGBSA dG Bind | Docking score | Glide gscore | MMGBSA dG Bind | Docking score | Glide gscore | MMGBSA dG Bind |
| 1 | -5.276 | -5.276 | -25.77 | -4.518 | -4.518 | -30.22 | -5.364 | -5.364 | -39.01 | -4.802 | -4.802 | -22.72 |
| **2** | **-5.907** | **-5.907** | **-66.50** | **-5.626** | **-5.626** | **-45.15** | -3.715 | -3.715 | -31.93 | -4.281 | -4.281 | -29.01 |
| 3 | -4.875 | -4.893 | - | -3.312 | -6.006 | -37.34 | -4.033 | -4.052 | -28.21 | -5.523 | -5.541 | -24.96 |
| 4 | -5.465 | -5.465 | -44.47 | -5.173 | -5.173 | -35.95 | -4.124 | -4.124 | -32.05 | -5.523 | -5.541 | -24.96 |
| **5** | **-5.624** | **-5.656** | **-47.34** | **-5.949** | **-5.98** | **-36.33** | -3.909 | -3.941 | -33.36 | -4.184 | -4.215 | -21.91 |
| 6 | -6.197 | -6.233 | -34.05 | -4.195 | -5.963 | -21.06 | -4.269 | -4.305 | -29.11 | -4.459 | -4.495 | -30.95 |
| 7 | -2.278 | -2.278 | - | -1.523 | -1.523 | -41.86 | 0.419 | 0.419 | -22.90 | -1.034 | -1.034 | -36.27 |
| 8 | -6.652 | -6.652 | -5.72 | -6.158 | -6.158 | -43.32 | -5.978 | -5.978 | -38.57 | -5.382 | -5.382 | -25.46 |
| 9 | -0.848 | -0.852 | - | 0.324 | 0.32 | -27.79 | 1.645 | 1.642 | -24.33 | 0.921 | 0.917 | -11.25 |
| 10 | -3.405 | -3.405 | - | -5.119 | -5.119 | -43.91 | -2.735 | -2.735 | -34.66 | -4.313 | -4.313 | -24.97 |
| 11 | -4.684 | -4.684 | - | -4.148 | -4.148 | -34.66 | - | - | - | -6.114 | -6.114 | -32.78 |
| 12 | -5.575 | -5.575 | -9.08 | -3.863 | -3.863 | -44.71 | -1.667 | -1.667 | -31.23 | -3.555 | -3.555 | -36.64 |
